# Supplementary material for: Complex computation from developmental priors
Source: Nat Commun. 2023 Apr 19;14:2226. doi: 10.1038/s41467-023-37980-1 (PMC10115783; doi:10.1038/s41467-023-37980-1)
Supplement: Supplementary file 1 — Supplementary Information [file 41467_2023_37980_MOESM1_ESM.pdf]

COMPLEX COMPUTATIONS FROM DEVELOPMENTAL PRIORS

SUPPLEMENTARY METHODS

A. Emergence of Cell Identity

To first approximation, a neuron's cell type (1) defines its connectivity and function, and (2) emerges from well-known factors, such as cell lineage and spatial gradients [32]. To make a machine learning analogy, the developmental patterning of cell types corresponds to a reproducible processing hierarchy, which we will assume to be a layered structure (Figure S1a). We can think of these as 'deterministic': if the organism develops normally, these cell identities emerge consistently and define the broad strokes of neurons' projections and connections [33]. However, neurons have also been shown to exhibit a random component to their wiring. For instance, stochastic alternative splicing of cell adhesion molecules allows for a significant amount of genetic variability to emerge [18, 30, 31]. Such processes further partition a single coarse-grained cell type, leading to divergent sub-identities, and thus divergent input and output profiles within a neural population.

Yet, the emergence of cellular variability is not completely random: the alternative splicing of implicated genes, and thus the resulting cell identity, has crucial biases in space and time [18, 29–31]. We can represent the expression of genes, or their alternative splices, as a mixture of gaussians, $G(\vec{x}|\mu_i, \sigma_i) \propto exp\{-\frac{1}{2}(\vec{x} - \vec{\mu}_i)^T \Sigma_i^{-1}(\vec{x} - \vec{\mu}_i)\}$, where G describes the expression level of a gene over a relevant parameterization of space and time ($\vec{x}$). Thus, the spectrum of "fine-grained" cell identities at a given location in the brain is dependent on the distribution of locally expressed genes (Figure S1b). What emerges is a definition of cell types that can be linked not only to a well-studied statistical learning paradigm, but is motivated by specific, highly profiled, genetic mechanisms.

At this point, we have arrived at developmentally motivated models of both cell identity and connectivity rules. As we meld these two components, we find that even simple neuronal applied to a small number of genes with spatial biases can produce complex circuits. Returning to the four genes in Figure S1, we can see that if a neuron only accepts connections from cells expressing blue genes, it will wire to cells in the top left corner of the space (Figure S1c, top left). A different neuron may select for the top center region by accepting connections from neurons expressing both blue and red genes (Figure S1c, top middle). As we extend these varying levels of selectivity, we find that we can partition the space in Figure S1c to form a convolutional tiling. Finally, if we incorporate "or" operators, we can produce more

specific filters on a given region (Figure S1d). Thus, spatially biased genes and simple wiring rules can begin to reproduce the inductive biases of the convolutional neural networks that underpin modern image recognition solutions.

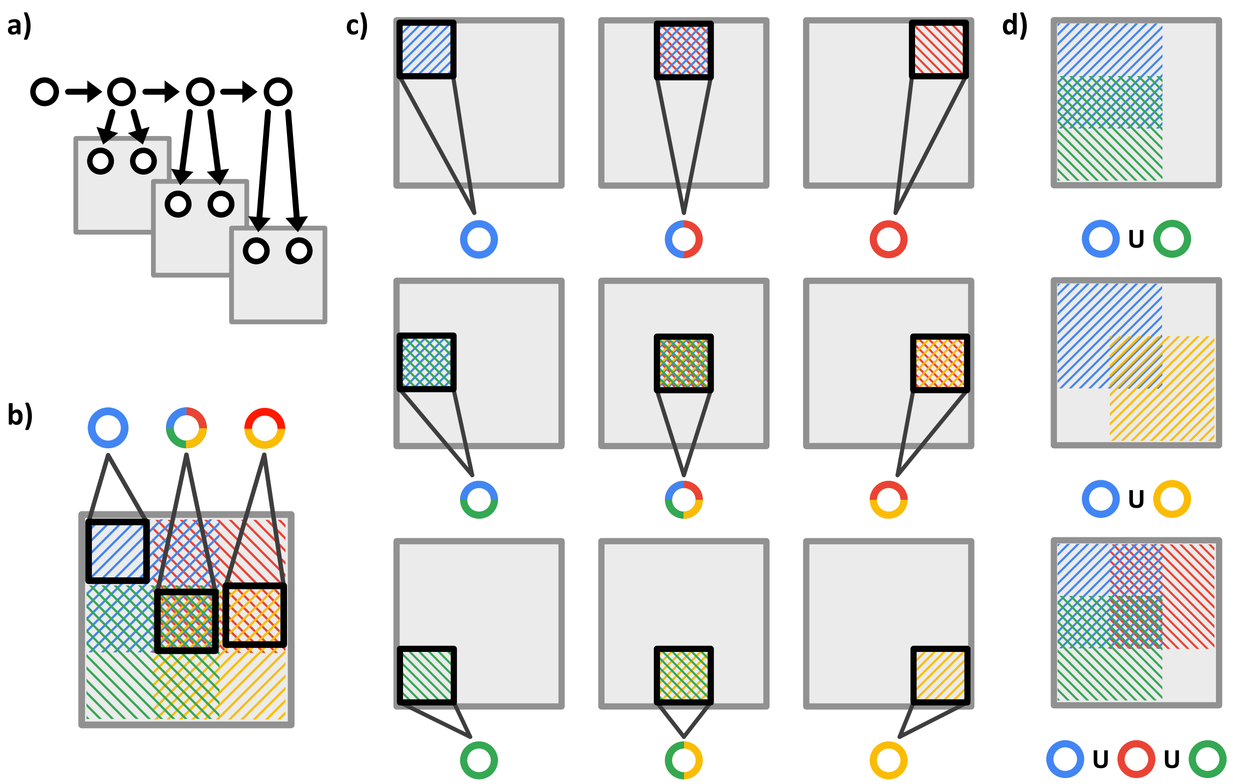

FIG. S1: **Developmental Priming of Computation.** **(a)** Coarse-grained cell types, such as those arising from lineage, can define a neuron's position in a processing hierarchy. **(b)** Spatial gene expression can produce diversity within the coarse-grained cell types. Here we have four genes (blue, red, green and yellow), each of which has a spatial bias to its expression (shading). When sampling from the top left, we find neurons that express the blue gene (left circle). However, if we sample at locations where the gene expression distributions overlap, we can find neurons with multiple genes expressed, such as red and yellow (right circle), or even all four genes (middle circle). **(c)** Four genes with spatial biases are sufficient for a spatial tiling to be developmentally primed. Neurons can have specific wiring rules, such as connecting to neurons that only expresses blue genes (top left). Other neurons may be more specific, incorporating "and" operators, for instance only receiving connections from neurons expressing both blue and red genes (top, center). **(d)** Incorporating "or" operators allows for the coding of spatial filters. Neurons that allow connections from blue or green genes can select for the left side of the region (top). A diagonal filter can be achieved by selecting for blue or yellow genes (middle). Finally, an upper triangle can be selected by being sensitive to blue, red, or green genes, but not yellow (bottom).

B. Transfer Learning

In addition to providing a developmental neuroscience inspiration for S-GEM, Figure S1 predicts that introducing biological biases to gene expression should produce recognizable structures in the networks' weights. When we observe the weights of a standard MLP trained on MNIST (Figure S2a), we find an uninspiring solution: the hidden nodes seem to learn outlines of numerals, such that the output nodes only have to "read out" the active solutions. In contrast, an MLP encoded by S-GEM exhibits spatial structure in the hidden layer (Figure S2b). We found that this spatial structure was unique to S-GEM, as it does not appear in GEM or Random Basis encodings (Figure S1). Yet, the spatial structure also emerges when S-GEM is used to encode a denoising autoencoder (Supplemental Methods, Figures S4 and S5) [37], highlighting the robustness of the phenomenon.

Given the similarity of the spatial weights of S-GEM to visual filters, we wondered if they contain generalized representations, akin to CNN layers. We tested for generalizability by asking our models to perform transfer learning, which quantifies whether representations learned by a neural network for one task can be efficient on a second task. For instance, we can train an MLP (Figure S2c, top left) on FMNIST, a categorization task for fashion items. Next, we can take the FMNIST-trained MLP and freeze $\mathbf{W_1}$, the weights between the input and hidden nodes (Figure S2c, top right), and train only $\mathbf{W_2}$, the weights between the hidden and output nodes, to solve MNIST. By freezing the first set of weights ($\mathbf{W_1}$), we freeze the way the network represents the FMNIST image in its hidden layer, and therefore ask it to perform on MNIST by learning a readout ($\mathbf{W_2}$) from this constrained "representation space." If the learned representation is a general computational solution (i.e. visual filters), it should be competitive on the new task. However, a standard MLP ($784 \times 784 \times 10$) performs poorly on task transfer, achieving less than 70% accuracy on MNIST after training on FMNIST (Figure S2d, top row), compared to the over 90% accuracy of an S-GEM or MLP trained solely on MNIST (Figure S2d, bottom two rows). The results are similar when the order of the tasks is reversed: an MLP trained first on MNIST, then trained with a frozen $\mathbf{W_1}$ on FMNIST performs at under 65% accuracy (Figure S2e, top row), compared to the above 80% accuracy by an S-GEM or MLP trained solely on FMNIST (Figure S2d, bottom two rows).

Given this baseline, we can test the performance of a neural network encoded by S-GEM (Figure S2c, bottom left) on transfer learning. Transfer learning through S-GEM can be done in two ways. On the one hand, we can freeze the spatial gene expression parameters

and the $\mathbf{O}$ matrix, and learn only the gene expression of the output nodes (thereby updating only $\mathbf{W_2}$, Figure S2c, bottom right), which we consider a model of evolutionary adaptations to novel visual environments. Alternatively, can use the weights encoded by S-GEM for $\mathbf{W_1}$, but update $\mathbf{W_2}$ directly through backpropagation, with no encoding (Figure S2c, top right), which we consider on-line transfer learning within an organism's lifetime. We find that both these approaches significantly outperform the transfer learning of the standard MLP, achieving 80-90% accuracy when going from FMNIST to MNIST (Figure S2d, rows 4 and 5) and 75-80% accuracy when going from MNIST to FMNIST (Figure S2e, rows 4 and 5). As a sanity check, we wanted to ensure that this significant improvement does not come from having introduced relevant spatial information through the genetic gradients. To test this, we initialized networks by S-GEM but did not train them on an initial task before freezing $\mathbf{W_1}$, and found that the resulting random representation (Figure S2d,e rows 2-3) outperformed standard MLP transfer accuracy, but did not achieve equivalent performance to the transferred S-GEM approaches. In summary, we find that the transfer learning capabilities of S-GEM are augmented by pertinent information in the spatial cell identities (Figure S3), but also leverage generalized representations developed through "evolution."

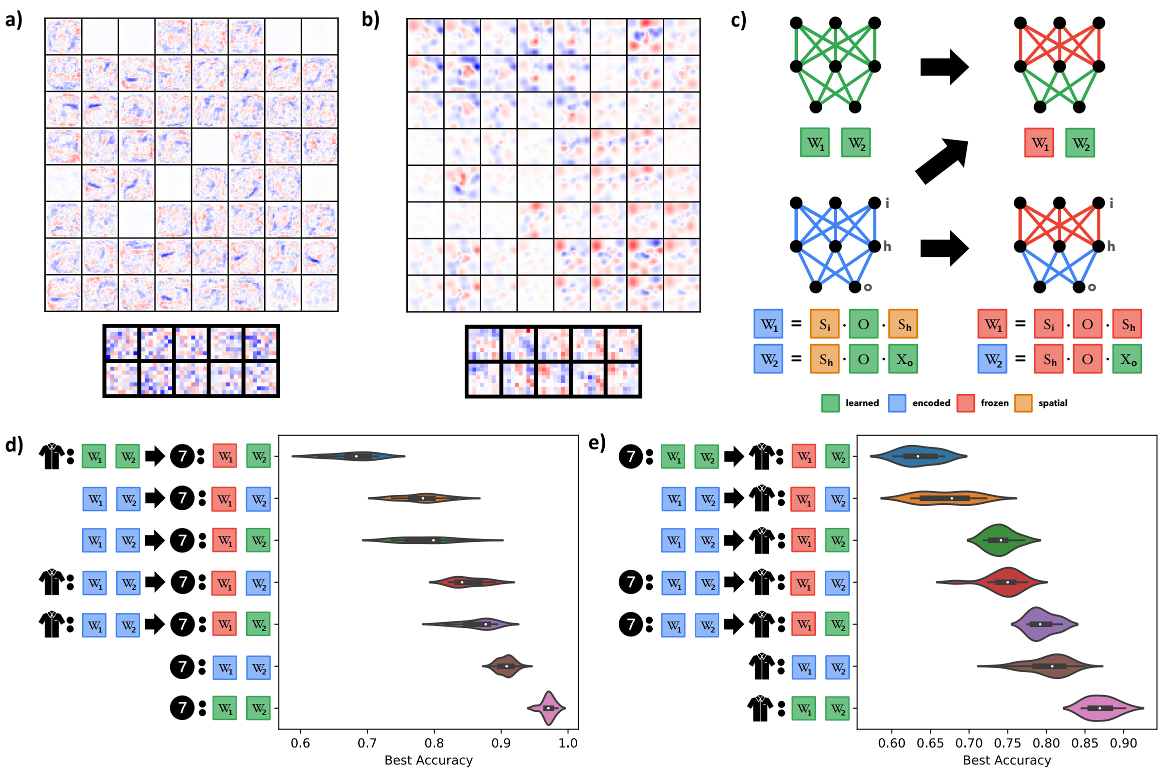

FIG. S2: **Transfer of Learned Representations.** **(a)** Weight matrices for a standard MLP (784 × 64 × 10) trained on MNIST. Each square shows the weight matrix of a single node, with inputs and nodes arranged spatially. For instance, the top left node of the hidden layer has weights of the MNIST shape 28×28, where the top left pixel in the box corresponds to the hidden node's weight from the top left pixel of the input image. **(b)** Weight matrices for an S-GEM (784 × 62 × 10) trained on MNIST, laid out as in (a). **(c)** Visualization of transfer learning. A standard MLP (Top, Left) can be trained on one task, e.g. FMNIST, then, with the first layer frozen (Top, Right), can be trained on a second task, e.g. MNIST. Alternatively, an ML-S-GEM (Bottom, Left) can be trained on a task, then can either be retrained to a new task by updating just the gene expression of the output layer (Bottom, Right) to model "Evolutionary Transfer," or the output weights can be retrained entirely (Top, Right) to model "Learning Transfer." **(d)** Violin plots (showing mean and quartile range, n = 10) for the performance on MNIST, with or without transfer learning. Top row: MLP is trained on FMNIST (shirt), then retrained on MNIST (number) with the first layer frozen. The next two rows consider architectures with weights initialized by ML-S-GEM, however the first layer was frozen immediately, and the resulting models were trained on MNIST by either learning the gene expression of the output layer (second row) or updating the hidden to output weights directly (third row). The next two rows train ML-S-GEM on FMNIST, and then are trained on MNIST by either learning the gene expression of the output layer (fourth row) or updating the hidden to output weights directly (fifth row). Last two rows contain an ML-S-GEM (sixth row) and standard MLP (last row) trained directly on MNIST. **(e)** Performance on FMNIST (mean and quartile range, n = 10), with or without transfer learning. Order of plots rows are akin to (d), however we are now transferring from MNIST to FMNIST.

C. MNIST Autoencoder Task

To test the possibility of hard-coding filters by modeling gene expressions as gaussians, we turn to an MNIST denoising autoencoder task that has been previously used to show the emergence of convolution by evolutionary optimization [37]. To begin, we set 10% of pixels in each MNIST digits to 0, and ask a network to reconstruct the original digit. We use a standard architecture [37] of 1 hidden layer with 100 neurons, and input and output layers of size $28 \times 28 = 784$ neurons (the size of an MNIST image). A sigmoid layer is included after the hidden layer and the output layer. For the spatial GEM approach, we place all the neurons on a grid (10×10 for hidden, 28×28 for input and output), and then pick G gaussians, with fixed σ but random μ, on the grid. This leads to the $N \times G$-sized $\mathbf{X}$ matrix being fixed as the value of the gaussian g_i at that neuron's location. We then train the $\mathbf{O}$ matrix, as well as any bias vectors. We compare this to a linear network and a standard GEM approach, where the $\mathbf{X}$ matrix can be learned. Training occurs on 5000 iterations of batches of size 64. We use a BCE loss, which compares the output image to the initial, unperturbed, MNIST digit.

We find that all three approaches can obtain a BCE of 0.001. However, the linear network requires 157,684 parameters, while the GEM can achieve competitive fitness with 10 genes, corresponding to 17,674 parameters. Finally, the spatial GEM can solve the task with 50 random gaussians, corresponding to only 5,884 trainable parameters, and is the only one of the three that evolves the weight structures determined by [37] to be reminiscent of convolution (Fig S4, S5).

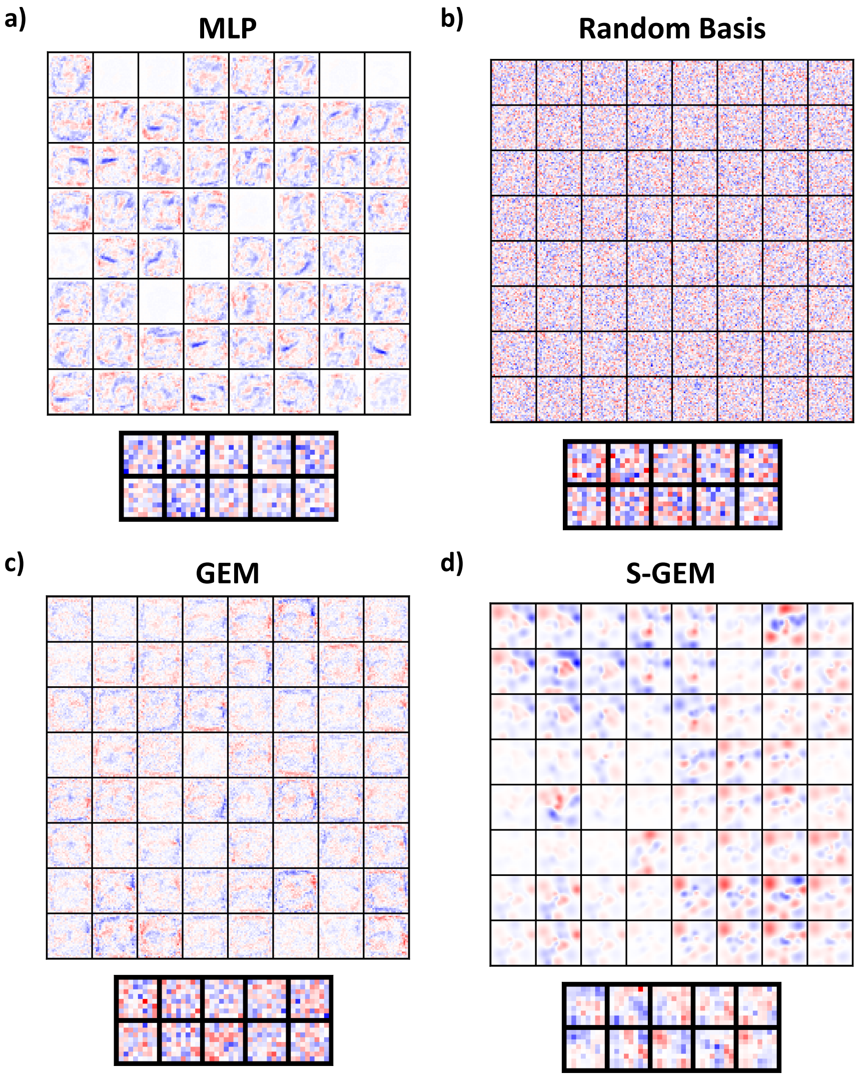

FIG. S3: **Weight Structure of an MLP Trained on MNIST.** **(a)** Weight matrices for a standard MLP ($784 \times 64 \times 10$) trained on MNIST. Each square shows the weight matrix of a single node, with inputs and nodes arranged spatially. For instance, the top left node of the hidden layer has weights of the MNIST shape 28×28, and the top left pixel in the box corresponds to the top left node in the input layer. **(b-d)** Weight matrices for **(b)** Random Basis, **(c)** GEM, and **(d)** S-GEM encodings of an MLP of size $784 \times 64 \times 10$ trained on MNIST, laid out as in (a).

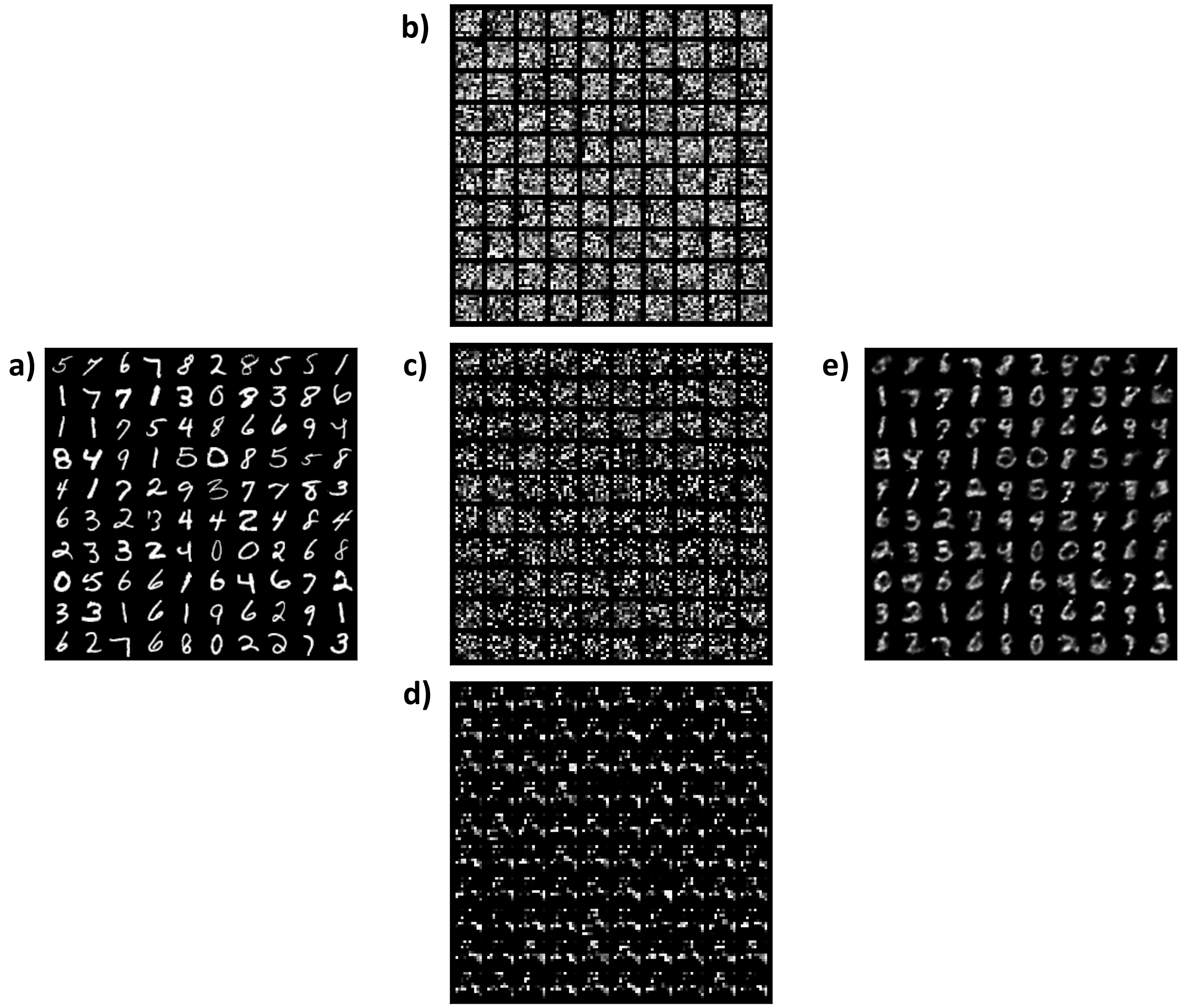

FIG. S4: **Visual Representation of MNIST Denoising Autoencoder Task.** **(a)** Visualization of standard MNIST digits used as input. **(b-d)** Representation of MNIST digits at hidden layer from **(b)** a linear network, **(c)** a standard GEM network, and **(d)** a spatial GEM network. The linear and GEM approaches have noisy, seemingly random, hidden representations, while the spatial GEM approach's representation recapitulates the spatial structure of the weights (Figure S2). **(e)** Sample output of the task.

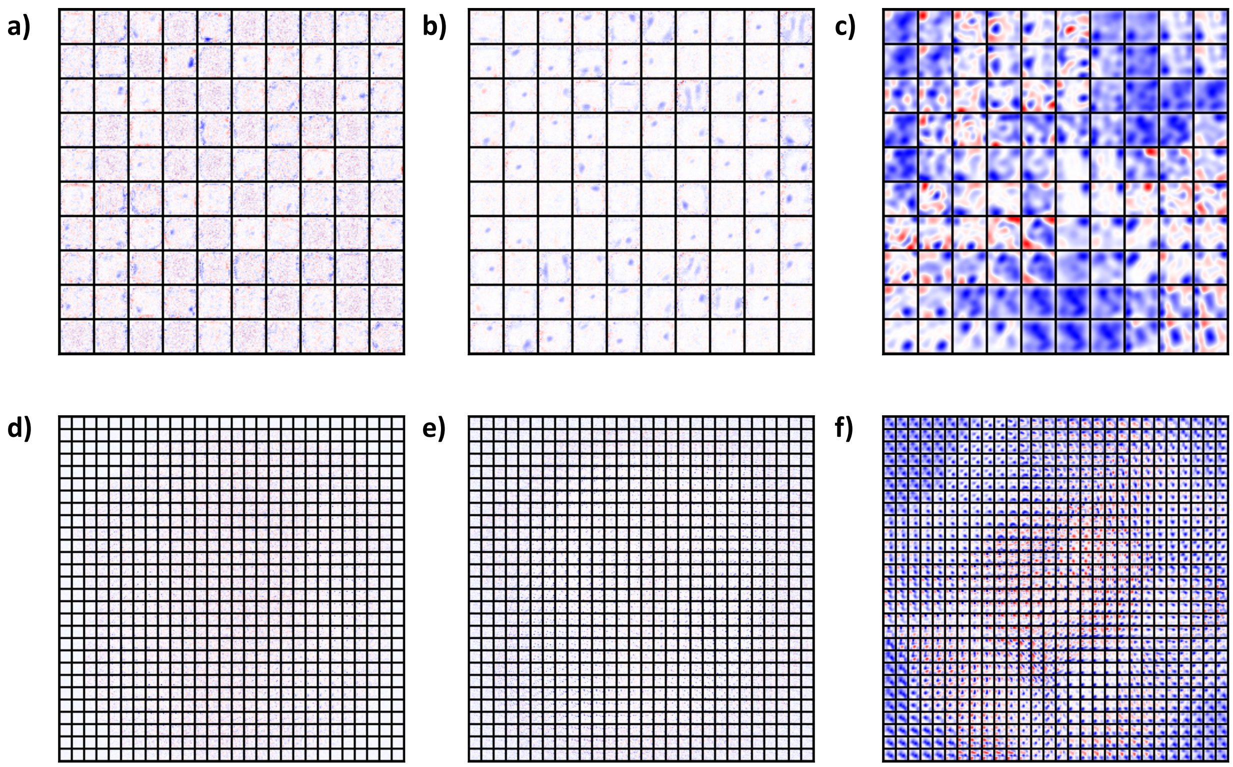

FIG. S5: **Visualization of MNIST denoising autoencoder weights.(a-c)** Hidden layer weights for **(a)** a standard linear network, **(b)** a standard GEM network, and **(c)** a spatial GEM network. Red corresponds to positive weights, white is near-zero, and blue is negative. While the spatial GEM network produces weight patterns akin to feature detectors, both the linear and standard GEM approaches learn weights that display less local similarity, both within and between neurons. **(d-f)** Output layer weights of the **(d)** linear, **(e)** GEM, and **(f)** spatial GEM networks. Again, the spatial GEM network displays apparent topographical structure, both within and between neurons, while the other two approaches learned heterogeneous weights.

D. Relation between GEM and PCA

In the case of an SLP parameterized by $W = X_1 O X_0^T$ where $\mathbf{X_1}$ is a fixed random matrix, the GEM encoding resembles a PCA where the first G basis vectors are kept. We do not consider this observation to be trivial, in fact, we find it compelling that cell-cell recognition rules (as captured in $W = X_1 O X_0^T$) can support PCA. This would indicate that the tuning of developmental gene expression levels supports a powerful evolutionary search for innate behaviors through a PCA parameterization.

On the other hand, the SLP example is primarily intended as a derivation and illustration

of the encoding approach, rather than a crucial result in itself. When extended to MLPs, $\mathbf{X}$s are shared from layer to layer (for instance: $\mathbf{X_1}$ is both the "output" identities of a first layer ($W_1 = X_0 O \mathbf{X_1}^T$) and the "input" identities of a second layer ($W_2 = \mathbf{X_1} O X_2^T$)). This sharing makes the learning process more complex than PCA, as the feature vectors of one layer become the projections of the next. While matrix decomposition has been previously used for encoding weight matrices [28], we abandoned this direction (e.g. $W = X_1 X_2^T$), as in work under preparation we found that the $\mathbf{O}$ proves important for creating a ruleset that can be applied, even when new layers or neurons are added to the network.

E. Structure of the ANN during training

Previous studies of the topological properties of ANNs [36] had shown that the modularity of a neural network varies over the course of training: initially stagnant (training batches 1-8, Figure S6a, 'Direct'), then increasing rapidly (batches 8-40), then slowly declining as the network fine-tunes (batches 40-10,000). This dynamics in modularity is reflected in comparisons to network accuracy, where a standard ANN saw an increase in modularity as accuracy increased up until 90%, after which modularity decreased as the network gained the final few percentage points of accuracy (Figure 6d).

We found that the relation between modularity and training to be more complex for GEM and S-GEM. The wiring rules of GEM promote a modular network (Figure S6b), as many rules act in concert, leading to dense connectivity with high modularity [23]. The dynamics of correctly parameterized (G = 10) or over-parameterized (G > 10) matches that of a normal ANN, although beginning higher and relaxing back to an initial level, rather than staying high with high accuracy (Figure 6b). It is interesting to note that under-parameterized GEMs (G = 3,5) have even higher modularity, but seem to be unable to restructure the network properly during training, as modularity stays stable, perhaps explaining why they are unable to attain high test accuracy. Yet, networks encoded by S-GEM do not show this pattern, retaining a high modularity throughout training, although networks with high G do display a small bump in the range of training batches 100-200 (Figure S6c). These patterns are reflected when we look at how modularity scales with accuracy, although it is interesting to note that the "bump" in GEM and S-GEM modularity occurs at a lower accuracy than for direct encoding (Figure S6d). Overall, we find that modularity is not the clearest metric of the network "coalescing" towards better performance.

In order to dig deeper, we also examined how the representation of direct and GEM

encoded neural networks evolve throughout training by studying the within and between class distances in PC space [36]. It was previously shown in standard ANNs that early training increases both within and between class distance, while late training acts to reduce within class distance, thereby developing more specific representation of the MNIST digits (Figure S7). As with the modularity results, the GEM encoding seems to follow the pattern of the direct encoding, except for the under-parameterized networks (G = 3, 5), where the within distance starts higher and stays higher than GEM networks that can properly solve the task (Figure S7b). In contrast, S-GEM encoded networks tend to increase their between and within distance later, with strange dynamics; rather than overshooting then lowering the within category distance, S-GEM encoded networks plateau to a fixed within distance, while overtraining then lowering between category distance. This unique dynamics may explain the success of S-GEM in meta-learning tasks, where the encoding trains slower, but coalesces to stable performance even under extreme compression.

For these experiments we largely reproduced the methods of [36], training a neural network with two hidden layers of 100 nodes on MNIST, although we used ADAM rather than SGD, and trained for only 10,000 batches, sampling the first 30; every 10 batches to 100; every 100 batches to 1,000; and every 1,000 batches to 10,000. We measure the modularity using networkx toolbox's community louvain script. We measure between and within distances based on the top 3 PCs of the hidden layer activations, as described in [36].

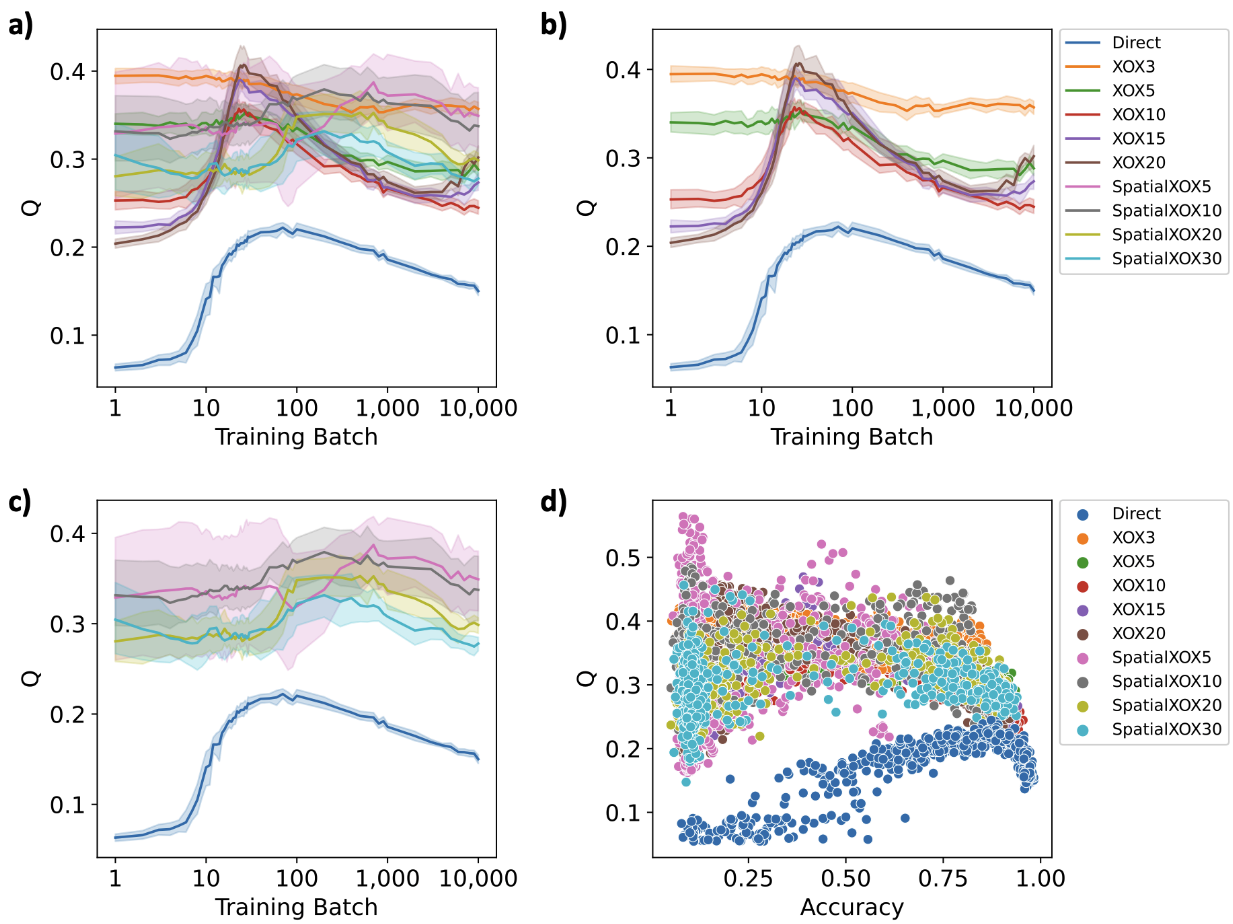

FIG. S6: **Modularity of ANNs under Training (a)** Modularity (Q) as a function of training batches of all conditions, with a focus on GEM conditions in **(b)** and S-GEM conditions in **(c)**. Lines indicate mean Q over 10 runs, with bands showing 95% confidence interval. Labels are shown next to (b), with the numbers denoting the number of genes provided for the GEM encodings. **(d)** Modularity (Q) as a function of network accuracy.

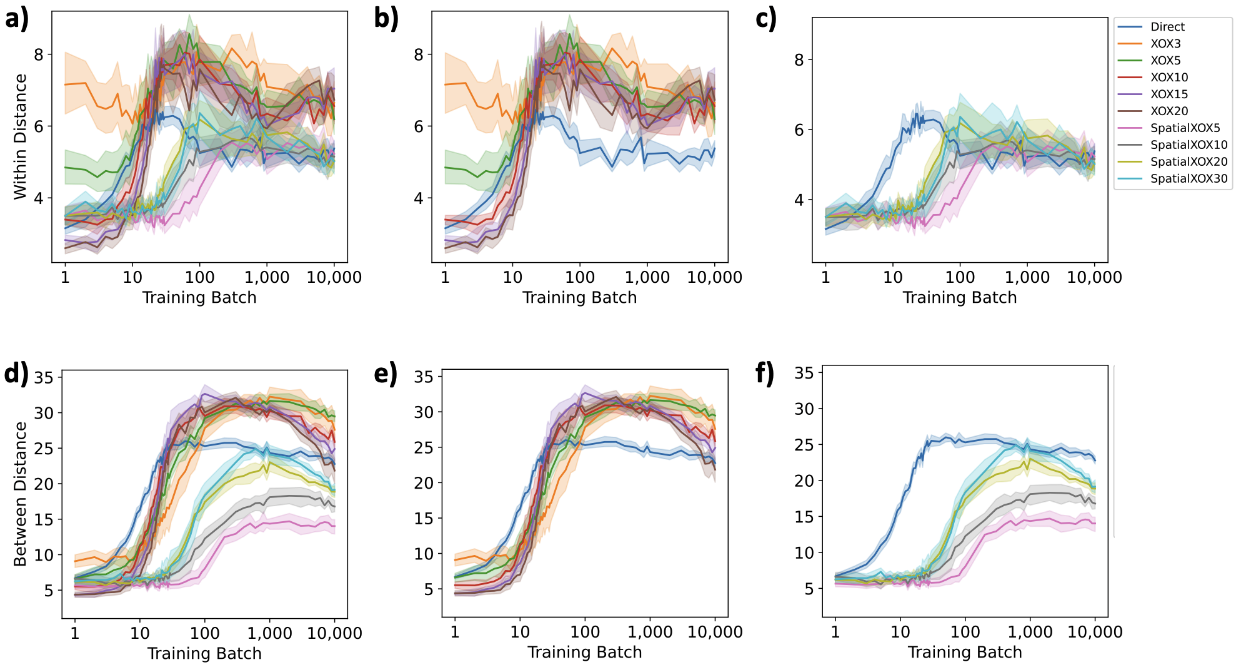

FIG. S7: **Representational Distances under Training (a)** Within-category distance given a PCA embedding of network activations for all conditions, with a focus on GEM conditions in **(b)** and S-GEM conditions in **(c)**. Lines indicate mean distance over 10 runs, with bands showing 95% confidence interval. Labels are shown next to (c), with the numbers denoting the number of genes provided for the GEM encodings. **(d)** Between-category distance given a PCA embedding of network activations for all conditions, with a focus on GEM conditions in **(e)** and S-GEM conditions in **(f)**. Colors and lines as in (a-c).
